# Naltrexone Prevents in Males and Attenuates in Females the Expression of Behavioral Sensitization to Ethanol Regardless of Maternal Separation

**DOI:** 10.3389/fendo.2016.00135

**Published:** 2016-10-18

**Authors:** Suzi E. Kawakami, Isabel M. H. Quadros, Deborah Suchecki

**Affiliations:** ^1^Department of Psychobiology, Escola Paulista de Medicina – Universidade Federal de São Paulo (UNIFESP), Sao Paulo, Sao Paulo, Brazil

**Keywords:** neonatal stress, locomotor sensitization, opioid system, alcohol

## Abstract

Maternal separation alters the activity of the opioid system, which modulates ethanol-induced stimulation and behavioral sensitization. This study examined the effects of an opioid antagonist, naltrexone (NTX), on the expression of behavioral sensitization to ethanol in adult male and female mice submitted to maternal separation from postnatal days (PNDs) 2 to 14. Whole litters of Swiss mice were either not separated [animal facility rearing (AFR)] or separated from their mothers for 3 h [long maternal separation (LMS)]. Starting on PND 90, male and female AFR and LMS mice received daily i.p. injections of saline (SAL) or ethanol (EtOH, 2.2 g/kg) for 21 days. Locomotor activity was assessed in cages containing photoelectric beams, once a week, to examine the development of behavioral sensitization. Five days after the end of the chronic treatment, animals were submitted to four locomotor activity tests spaced by 48 h, to assess the expression of behavioral sensitization. In all tests, animals received two i.p. injections with a 30-min interval and were then assessed for locomotor response to different treatment challenges, which were: SAL/SAL, SAL/EtOH (2.2 g/kg), NTX 2.0 mg/kg (NTX2)/EtOH, and NTX 4.0 mg/kg (NTX4)/EtOH. Regardless of maternal separation, EtOH-treated male and female mice displayed increased locomotor responses to EtOH during the 21-day treatment, indicating the development of behavioral sensitization. In the SAL/EtOH challenge, EtOH-treated LMS and AFR male and female mice exhibited higher locomotor activity than their SAL-treated counterparts, indicating the expression of sensitization. The coadministration of either dose of NTX blocked the expression of locomotor sensitization in both AFR and LMS male mice with a history of EtOH sensitization. In females, a significant attenuation of EtOH sensitization was promoted by both NTX doses, while still maintaining an augmented stimulant response to EtOH. Importantly, maternal separation did not interfere in this phenomenon. These results indicate that expression of behavioral sensitization was importantly modulated by opioidergic mechanisms both in male and female mice and that maternal separation did not play a major role in either development or expression of this EtOH sensitization.

## Introduction

Maternal care is essential for the proper development of altricial mammals, whose central nervous system maturation takes place postnatally. The ontogenesis of the hypothalamic–pituitary–adrenal (HPA) axis stress response also occurs during the first weeks of life in mice and rats and is regulated by maternal presence, which tonically inhibits its activation by most stressors. Maternal care is responsible for maintaining the pups’ HPA axis quiescent; specifically stroking of the anogenital area inhibits the ACTH stress response, whereas lactation reduces corticosterone secretion (1–5). This inhibition is demonstrated by separating the offspring from its mother for periods of 8–24 h, resulting in elevated stress-induced ACTH and corticosterone stress responses (2, 4, 6–8).

Disruption of the mother–infant relationship produces long-term alterations in numerous behaviors and brain systems (9), including vulnerability to drug abuse (10) and changes in the activity of the opioid system (11). A considerable number of studies have shown that separation of pups from their mothers for long periods of time during the first 2 weeks of life [3–6 h/day; hereby referred to as long maternal separation (LMS)] affects brain opioid levels (12–15). Increases in immunoreactivity for Met-enkephalin peptides are detected in regions associated with reward and emotional behaviors, including the medial prefrontal cortex (12, 13), while less consistent changes are found in dynorphin-B levels, depending on the brain region and separation protocol (11). Additionally, LMS animals show greater sensitivity to morphine, an opioid agonist (16), compared to control animals. Changes in the opioid system are particularly relevant within the context of drug abuse, since these neuropeptides are involved in motivation and reward, regulating the activity of the dopaminergic mesolimbic system by means of μ-, δ-, and κ-opioid receptors (17–19). However, the effects of LMS on drug addiction-related behaviors appear to be sexually dimorphic for it increases self-administration of psychostimulants (10), morphine (20), and ethanol in males (21–24), but not in females (13, 25). LMS also modifies behavioral sensitization to cocaine (26) and ethanol in females, but not in males (27), indicating a strong influence of sex and paradigm used to evaluate the neurobiological aspects involved in drug addiction.

Locomotor sensitization is defined as an augmented behavioral response, e.g., locomotor activity, to the stimulant effects of drugs upon repeated administration. This paradigm has been used to study neuroadaptive changes induced by chronic EtOH administration, which may contribute to EtOH addiction. Interestingly, opioids seem to play a key role in the motivational aspects of drug and alcohol abuse in several animal models, including behavioral sensitization [for reviews, see Ref. (28, 29)]. Non-selective opioid receptors antagonists, such as naltrexone (NTX) or naloxone, decrease EtOH-induced stimulant effect (30, 31) and inhibit the development of behavioral sensitization to ethanol (32, 33). However, the expression of behavioral sensitization to EtOH is not affected by these opioid receptor antagonists (32, 34). In the present study, we evaluated the effect of NTX, a non-selective opioid antagonist of important clinical value in the treatment of alcohol dependence [for reviews, see Ref. (18, 35)], on the expression of behavioral sensitization to ethanol in maternally separated adult mice. For this, we employed higher NTX doses than previous studies (32), and also tested both male and female mice to investigate possible sex differences [as opposed to only testing males, as in Ref. (32–34)]. Additionally, we tested the effect of an early life stress manipulation, which could further modulate the expression of ethanol sensitization (26, 27) and the sensitivity to NTX effects (11–16).

## Materials and Methods

### Animals

Swiss mice were mated in the animal facility of the Department of Psychobiology and daily inspected for the presence of pups. The day of the birth was designated postnatal day (PND) 0. On PND 1, litters were culled to 5 males and 5 females. Animals were maintained in a controlled 12-h light–dark cycle (lights on at 7:00 a.m. and off at 7:00 p.m.) and temperature (23 ± 2°C). Food and water were provided *ad libitum* throughout the entire study. Animal manipulations and protocols were approved by the Ethics Committee in Research (CEP# 521/07), and the experiments were carried out in accordance with Brazilian regulations on the use and care of animals.

### Neonatal Manipulations

From PND 2 to 14, pups were subjected to daily maternal separation for 180 min (LMS) or not separated until weaning on PND 22 [animal facility rearing (AFR)]. In the LMS group, whole litters were removed from the nest, at ~12:00 hours, and placed in separate cages on a heating pad set at 33°C, whereas the mothers remained in the home cage and, at the end of the allotted period, litter and mother were reunited in the home cage. Once a week during the separation, half of the old bedding material was mixed with clean material, in order to prevent excessive ammonia accumulation and still keeping olfactory familiarity from the old bedding material. AFR litters were handled during cage cleaning (three times a week). Weaning took place on PND 22 and two to three litters from the same group were housed in plastic cages (10–15 animals).

### Drugs

Ethanol (Synth) was prepared fresh every day, in a concentration of 15% w/v, in 0.9% saline (SAL) and administered at a dose of 2.2 g/kg (i.p.). The opioid antagonist, NTX (Sigma-Aldrich) was also prepared before use, in 0.9% SAL, and the doses were chosen based on a previous study (33).

### Behavioral Sensitization

#### Habituation (Hab)

At PND 90, AFR and LMS male and female mice (8–9 litters/manipulation) were individually tested in Opto-Varimex activity cages (Columbus Instruments, Columbus, OH, USA), which detect locomotion by interruptions of horizontal photoeletric beams, for 15 min.

#### Development Phase

**Figure 1:**
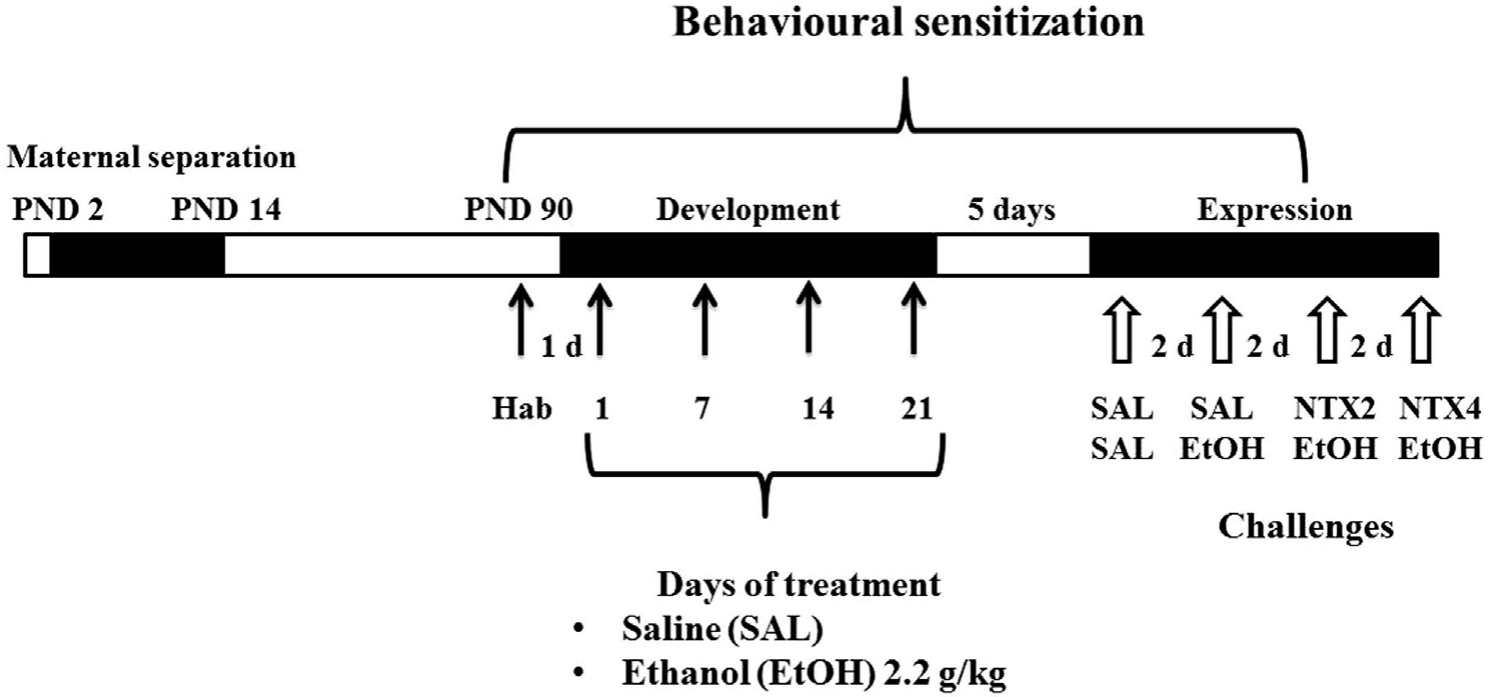
**Schematic representation of the experimental design**. Black horizontal bars represent daily saline (SAL) or ethanol (EtOH) i.p. administration. The numbers represent the locomotor activity tests. White arrows represent two i.p. administrations, first with SAL or NTX (2.0 or 4.0 mg/kg) and second with SAL or EtOH.

Fourty-eight hours after the habituation test, the animals were allocated into two treatment groups, and received daily injections of SAL or 2.2 g/kg EtOH, i.p, for 21 days (*n* = 10–13 animals/sex/group), since studies from our lab have shown that this treatment induces consistent levels of behavioral sensitization (36, 37) (Figure 1). Locomotor activity was measured once a week (days 1, 7, 14, and 21 of treatment), immediately after the treatment, for 15 min.

#### Expression Phase

Five days after the last administration of the development/induction phase, animals were submitted to 4 challenges spaced by 48 h. In all challenges, animals received two i.p. injections spaced by 30 min (Figure 1). All mice were submitted to drug challenges in the following order: SAL/SAL, SAL/EtOH (2.2 g/kg), NTX (2.0 mg/kg)/EtOH (2.2 g/kg), and (NTX, 4.0 mg/kg)/EtOH (2.2 g/kg). Immediately after the second administration, the animals were placed in the activity cages, and locomotor activity was measured for 15 min. All procedures were carried out in the afternoon (between 12:00 and 17:00 hours).

### Statistical Analysis

Locomotor response to habituation was compared between groups (AFR, LMS) by Student’s *t-*test. The locomotor response during the development phase was analyzed by three-way analysis of variance (ANOVA) with group (AFR, LMS), treatment (SAL, EtOH), and day (repeated measure) as main factors. During the expression phase, the locomotor response was analyzed by a three-way ANOVA for repeated measures, with group (AFR, LMS), pretreatment (SAL, EtOH), and challenge as the repeated measure (Sal/Sal, Sal/EtOH, NTX2/EtOH, and NTX4/EtOH). Males and females were analyzed separately. When appropriate, *post hoc* analysis was carried out using Newman–Keuls test, with the level of significance set as *p* ≤ 0.05.

## Results

### Habituation

Pairwise comparison showed no differences in locomotion between LMS and AFR in either male [*t*_(46)_ = 0.31; *p* > 0.05] or female mice [*t*_(47)_ = 1.51; *p* > 0.05] (Table 1).

**Table 1:** **Locomotor activity (counts), during habituation, of male and female mice kept with their mothers for the entire developmental period [animal facility rearing (AFR)] or submitted to long maternal separation (LMS), from postnatal days 2 to 14**.

	Males	Females
AFR	1209.57 ± 369.82 (23)	1573.75 ± 523.22 (24)
LMS	1183.04 ± 212.20 (25)	1375.68 ± 387.89 (25)

*The values are presented as mean ± SD. Number of animals/group is shown in parenthesis*.

### Development of Behavioral Sensitization

#### Male Mice

**Figure 2:**
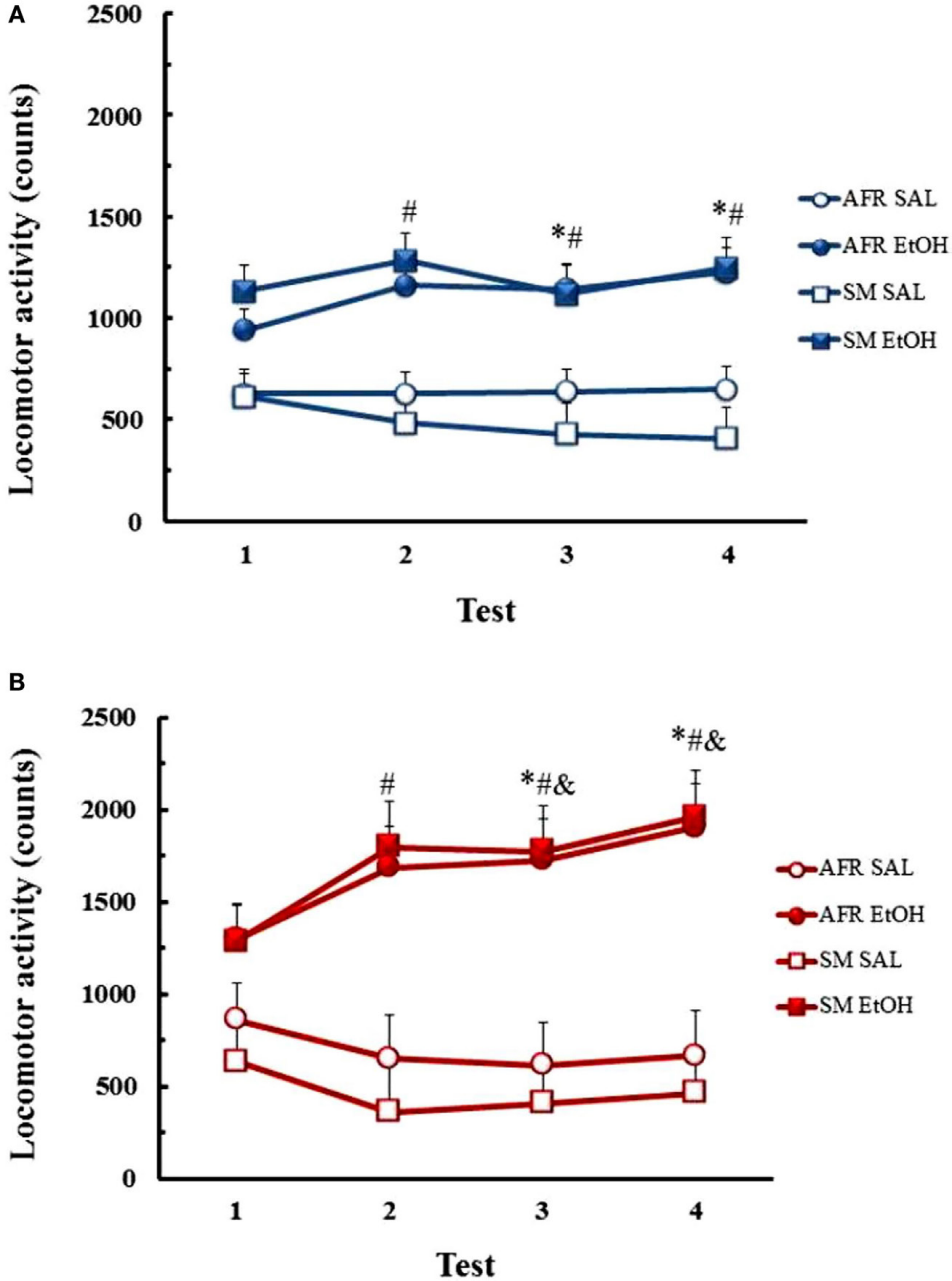
**Locomotor activity counts (mean ± SEM) throughout the course of saline (SAL) or ethanol (EtOH) chronic treatment in AFR and LMS male (A) and female mice (B)**. Number of animals/group for each condition (sex, group, treatment) was 10–13. * – Different from saline-treated groups; # – different from day 1; & – different from day 7.

ANOVA revealed main effects of treatment [*F*_(1,44)_ = 25.41, *p* < 0.01] and day [*F*_(3,132)_ = 4.36, *p* < 0.01] and an interaction between treatment and day [*F*_(3,132)_ = 10.32, *p* < 0.01], with no differences between AFR and LMS manipulations (Figure 2). Newman–Keuls tests for the treatment × day interaction showed that EtOH-treated mice presented increases in locomotor activity on test days 7, 14, and 21, relative to SAL-treated animals (*p* < 0.05). EtOH-induced hyperactivity was also higher on days 14 and 21, when compared to responses to EtOH on days 1 and 7 (*p* < 0.01).

#### Female Mice

ANOVA revealed main effects of treatment [*F*_(1,45)_ = 45.80, *p* < 0.01] and day [*F*_(3,135)_ = 28.01, *p* < 0.01] and interaction between these factors [*F*_(3,135)_ = 22.85, *p* < 0.01], with no group effects (AFR vs. LMS). Analysis of the interaction showed that the locomotor activity of EtOH-treated mice was higher on days 7, 14, and 21 than on day 1 of treatment (*p* < 0.01) and on days 14 and 21 compared to day 7 (*p* < 0.01). EtOH-treated mice presented higher locomotor activity than SAL-treated mice on days 7, 14, and 21 of treatment (*p* < 0.01).

### Expression of Behavioral Sensitization

#### Males

**Figure 3:**
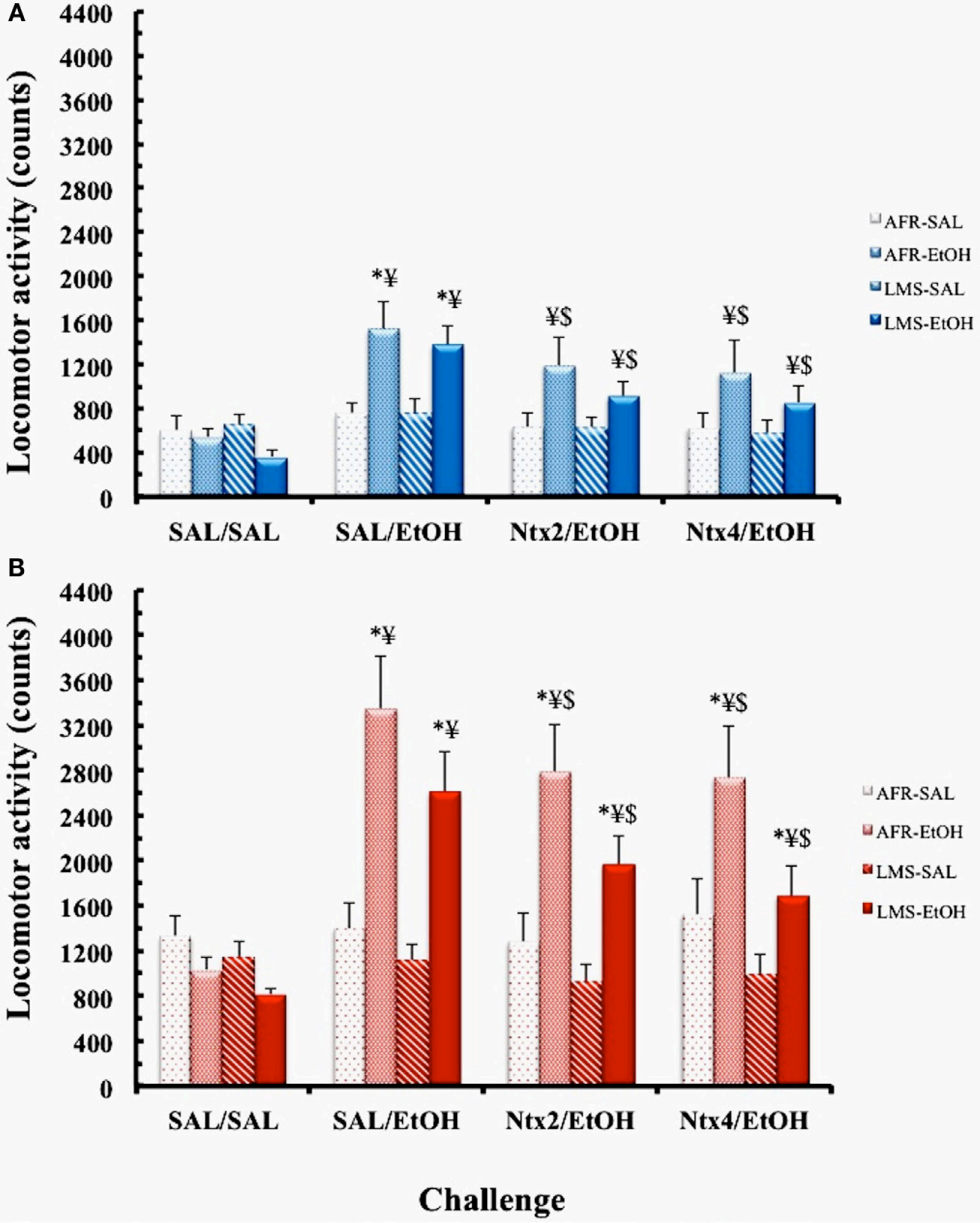
**Locomotor activity counts (mean ± SEM) of saline (SAL)- or ethanol (EtOH)-pretreated AFR and LMS males (A) or females (B) in the challenges SAL/SAL, SAL/EtOH, naltrexone 2.0 mg/kg – NTX2/EtOH, and naltrexone 4.0 mg/kg – NTX4/EtOH**. EtOH dose was 2.2 g/kg (i.p.). Number of animals/group for each condition (sex, group, treatment) was 10–13. * – different from SAL-treated groups; ¥ – different from SAL/SAL challenge, $ – different from SAL/EtOH challenge.

Three-way ANOVA revealed main effects of pretreatment [*F*_(1,44)_ = 5.6872, *p* < 0.03] and challenge [*F*_(3,132)_ = 19.841, *p* < 0.00001] and an interaction between these factors [*F*_(3,132)_ = 12.395, *p* < 0.00001], with no group effect (Figure 3). *Post hoc* analysis of the interaction showed that mice with a history of EtOH treatment displayed higher locomotor activity than SAL-pretreated counterparts in the SAL/EtOH challenge (*p* < 0.0005). Such differences were no longer observed during the NTX2/EtOH and NTX4/EtOH challenges, suggesting that EtOH-induced expression of sensitization was prevented by NTX. Moreover, in mice with a previous history of EtOH treatment, locomotor response to EtOH was the highest during the SAL/EtOH challenge, with significant reductions when NTX was administered with EtOH (NTX2/EtOH and NTX4/EtOH challenges, *p*’s < 0.005). In SAL-pretreated male mice, no changes in locomotor response were observed with any of the drug challenges, relative to the SAL/SAL challenge.

#### Females

A three-way ANOVA showed main effects of group [*F*_(1,45)_ = 4.303; *p* < 0.05], pretreatment [*F*_(1,45)_ = 12.983; *p* < 0.001], and challenge [*F*_(3,135)_ = 22.48; *p* < 0.00001] and an interaction between these two latter factors [*F*_(3,135)_ = 23.14; *p* < 0.00001]. Newman–Keuls analysis of the overall group effect showed that LMS animals exhibited lower locomotor activity than AFR mice (*p* < 0.03). Analysis of the pretreatment × challenge interaction revealed that mice with a history of EtOH preexposure displayed higher locomotor activity than SAL-pretreated ones in the SAL/ETOH (*p* < 0.0005), NTX2/EtOH (*p* < 0.001), and NTX4/EtOH (*p* < 0.005) challenges. However, the sensitized response to EtOH was significantly attenuated by co-treatment with both doses of NTX (NTX2 or NTX4; *p*’s < 0.005). In mice pretreated with SAL, no changes in locomotor behavior were induced by challenges with EtOH or NTX/EtOH coadministration.

## Discussion

The results of the present study showed that the non-selective opioid antagonist, NTX, blocked the expression of behavioral sensitization to EtOH in male mice, and attenuated this phenomenon in females, regardless of the neonatal manipulation. Moreover, maternal separation had no impact on either the development or the expression of EtOH sensitization. Both males and females showed significant augmentation of locomotor responses to EtOH during the 21-day treatment (development of sensitization) and maintained a sensitized stimulant response when challenged with EtOH during the expression tests.

In previous studies from our group, repeated EtOH administration induced behavioral sensitization in mice (36–39). Although an initial study reported that LMS could accelerate the development of EtOH sensitization in female, but not male mice (27), this was not confirmed using a more robust, 21-day treatment protocol (39), as replicated in our current findings. Thus, the facilitatory effects of LMS on EtOH sensitization seem to only emerge with weaker sensitizing regimens (e.g., 5 EtOH injections), and in females. However, EtOH-induced corticosterone responses were higher in chronically EtOH-treated LMS males than in EtOH-treated controls, with no changes observed in females (39). Such changes in corticosterone response to chronic EtOH were not observed when mice received fewer EtOH treatments (27). Thus, LMS may modulate different behavioral and physiological/hormonal responses to EtOH in a sex-dependent and exposure-dependent manner.

A role for opioid receptor modulation of acute EtOH locomotor stimulation was reported by studies showing that opioid antagonists reduced EtOH-induced hyperactivity in mice (30–32), despite controversial findings (40). Coadministration of non-specific opioid receptor antagonists, such as NTX and naloxone, blocked the development of EtOH locomotor sensitization (32, 33). However, in mice previously sensitized to EtOH, NTX and naloxone failed to prevent the expression of EtOH sensitization (32, 34). In contrast, the present study showed important effects of NTX preventing and/or attenuating the expression of EtOH sensitization in males and females, while not inducing locomotor effects in animals with no previous EtOH history. This finding contrasts with Abrahao and colleagues, who reported no effect of NTX on the expression of EtOH sensitization (34). However, the NTX dose was considerably lower in that study, 0.1 mg/kg (34). Indeed, in a pilot study using male mice with no neonatal manipulation, we observed significant reduction of EtOH sensitization using NTX doses of 1 mg/kg and higher, but not with a 0.5 mg/kg dose (data not shown). The dose range necessary to block the expression of sensitization in male mice in our study (2 mg/kg) was similar to that required for preventing the development of EtOH sensitization as reported by Pastor and Aragon [1 or 2 mg/kg (33)].

Despite acting as non-selective antagonists at opioid receptors, both NTX and naloxone have preferential effects on μ-receptors, rather than on δ-receptors (30, 33, 41). Thus, our findings support an additional role for μ-receptors in the modulation of the expression of EtOH sensitization, besides mediating the development of EtOH sensitization (32, 33). Indeed, Pastor and Aragon showed that both NTX and a selective antagonist at μ-receptors, CTOP, blocked the development of EtOH sensitization, which was not affected by a delta-receptor antagonist, naltrindole. Moreover, the facilitation of EtOH sensitization after a period of EtOH consumption was absent in a recombinant line of mice with reduced expression and function of μ-receptors, CXBK mice (42). Altogether, these studies point to μ-receptors as the critical target for NTX’s effects on EtOH sensitization. A putative mechanism for NTX effects relies on the modulation of dopamine neurons projecting from the ventral tegmental area (VTA) to the nucleus accumbens (NAcc), a pathway involved in drug-stimulation, sensitization, and reward [e.g., Ref. (18, 19, 43)]. Opioid receptors, including μ-receptors, are located in both regions, where they modulate dopamine output directly (in the NAcc) or indirectly (via GABA interneurons in VTA) (18, 19). Indeed, local administration of NTX into the VTA or the NAcc inhibits acute EtOH-induced stimulation in mice (29). Thus, it would be expected that μ-receptor blockade in either or both brain regions could participate in the prevention/attenuation of the expression of EtOH sensitization reported in this study.

Remarkably, while NTX treatment blocked the expression of EtOH sensitization in males, in females there was only an attenuation of EtOH-sensitized response. In males, NTX administration, at both doses, blocked the expression of behavioral sensitization, since there were no longer differences in locomotor behavior between SAL- and EtOH-pretreated mice in the challenges. In females, both doses of NTX reduced, but failed to completely prevent a sensitized response to EtOH, suggesting a sexual dimorphism in the behavioral response to this opioid antagonist. In agreement with these findings, several studies report on sexual differences in response to opioidergic drugs. For example, NTX treatment is less effective in women than in men (44, 45), men are more vulnerable to opioid addiction than women (46), and males are more responsive to analgesic drugs than females, in several species (47). In a recent study carried out with rats bred for increased preference for ethanol, a low acute dose of NTX was effective to block spontaneous ethanol intake in male, but not in female, rats (48). NTX is also capable to reduce the intake of a highly palatable sucrose solution only in LMS males, but not in females (49). Sex steroid hormones appear to regulate the density of opioid receptors in the hypothalamus, with increased density of μ-opioid receptors during proestrus, and changes in μ receptor density in other limbic regions induced by hormone replacement in ovariectomized rats (50). Interestingly, full agonists of μ receptors in males act as partial agonists in female rats and primates (51), suggesting smaller affinity for these receptors in females. Thus, the reduced efficiency of NTX in blocking the expression of EtOH sensitization in female mice may be due to a smaller affinity/efficacy in μ-opioid receptor signaling in females.

In the present study, the only effect of maternal separation was seen in female mice, which displayed lower locomotor activity than their AFR counterparts during the expression, but not during the development of behavioral sensitization. Few studies had compared the induction and/or expression of behavioral sensitization between maternally separated male and female animals, with contradictory results. LMS has been reported to increase induction of behavioral sensitization to cocaine in male and female mice, but only males exhibited increased expression to a cocaine challenge (26). As mentioned in the Introduction, development of EtOH sensitization was facilitated in LMS female, but not in male mice, using a weaker sensitizing protocol (27), but not with a stronger one, with more prolonged EtOH treatment (39). Amphetamine sensitization was also not modified by maternal separation in rats (52, 53). However, LMS reduces the response rate for intracranial self-stimulation in female, but not in male rats (54).

In conclusion, NTX blocked the expression of EtOH-induced behavioral sensitization in male mice, while significantly attenuating EtOH sensitization in females, with no effects of neonatal manipulation. The only detectable effect of maternal separation was an overall reduced locomotor behavior of female mice during the expression tests for EtOH sensitization.

## Author Contributions

SK planned and performed the experiments described, analyzed the results, and wrote the original version of the manuscript. SK, IQ, and DS participated in defining experimental design, data analysis, and interpretation of results, as well as revising and writing the final version of the manuscript.

## Conflict of Interest Statement

The authors declare that the research was conducted in the absence of any commercial or financial relationships that could be construed as a potential conflict of interest.
